# Health-Related Quality of Life and Survival in Metastasized Non-Small Cell Lung Cancer Patients with and without a Targetable Driver Mutation

**DOI:** 10.3390/cancers13174282

**Published:** 2021-08-25

**Authors:** Nicole E. Billingy, Vashti N. M. F. Tromp, Corina J. G. van den Hurk, Annemarie Becker-Commissaris, Iris Walraven

**Affiliations:** 1 Department of Pulmonology, Amsterdam UMC, Cancer Center Amsterdam, Amsterdam Public Health Research Institute, 1081 HV Amsterdam, The Netherlands; n.billingy@amsterdamumc.nl (N.E.B.); a.becker@amsterdamumc.nl (A.B.-C.); 2 Department of Clinical Pharmacology and Pharmacy, Amsterdam UMC, Amsterdam Public Health Research Institute, 1081 HV Amsterdam, The Netherlands; v.tromp@amsterdamumc.nl; 3 Department of Research, Netherlands Comprehensive Cancer Organisation (IKNL), 3511 DT Utrecht, The Netherlands; c.vandenhurk@iknl.nl; 4 Department for Health Evidence, Radboud University Medical Center, 6500 HB Nijmegen, The Netherlands

**Keywords:** non-small cell lung cancer, metastatic lung cancer, health-related quality of life, targetable driver mutation

## Abstract

**Simple Summary:**

NSCLC patients with a targetable driver mutation are entitled to receive targeted therapy. Targeted therapy is usually less toxic than chemotherapy and immunotherapy. However, low-grade side effects are common and often chronic, which could also negatively impact a patient’s health-related quality of life (HRQOL). HRQOL in NSCLC patients with and without a targetable driver mutation is thus likely to be different. To optimize clinical decision making for individualized treatment in patients with metastatic NSCLC, it is necessary to understand differences in HRQOL and survival outcomes between patients with and without a targetable driver mutation. In this study, we show that NSCLC patients with a targetable driver mutation have favorable HRQOL over time compared to patients without a targetable driver mutation. Furthermore, clinically relevant HRQOL declines over time were significantly associated with worse survival. HRQOL can therefore play an important role in shaping patients’ expectations of their prognosis.

**Abstract:**

Background: The aim of this study is to compare long-term health-related quality of life (HRQOL) and survival in metastatic NSCLC patients with (M+) and without (M−) a targetable driver mutation. Methods: An observational study was performed within the prospective SYMPRO-lung study (NL7897). HRQOL questionnaires were completed at baseline, 15 weeks, and 6 months. Generalized estimating equations (GEE) were used to assess clinically significant declines in HRQOL (>10 points) over time. Kaplan–Meier survival curves were plotted for both progression-free survival (PFS) and overall survival (OS). Results: 81 metastatic NSCLC patients were included (M+ patients; 16 (20%)). M+ patients had a significantly better global HRQOL (mean difference 12.8, ES 0.61), physical functioning (mean difference 13.4, ES 0.63), and less appetite loss (mean difference 23.1, ES 0.73) at 15 weeks of follow-up compared to M− patients. Patients with a clinically relevant decline in HRQOL at 6 months of follow-up had a significantly shorter PFS (5 months vs. 12 months, *p*-value < 0.001) and OS (11 months vs. 16 months, *p*-value 0.002). Conclusions: M− NSCLC patients have less favorable HRQOL over time compared to M+ patients. Furthermore, clinically relevant HRQOL declines over time were significantly associated with worse survival. HRQOL can therefore play an important role in in shaping patients’ expectations of their prognosis.

## 1. Introduction

Lung cancer is the second most commonly diagnosed cancer worldwide, with an estimated incidence of 2.2 million patients each year. Lung cancer is thereby the leading cause of cancer-related mortality with 1.8 million deaths yearly [1]. Eighty-four percent of all lung cancers are non-small cell lung cancer (NSCLC), and of those, half of patients are diagnosed when metastases are already present [2]. 

For metastatic NSCLC patients, treatment is aimed at life extension while improving or at least preserving health-related quality of life (HRQOL). Due to the increased availability of treatments, especially when a targetable driver mutation is present, treatment decisions in metastatic NSCLC have become a delicate and complicated process for both the clinician as well as the patient and their relatives [3,4]. Currently, most clinical decisions are based on ‘traditional’ clinical criteria, such as mutational status and TNM stage. These clinical criteria might lead to choosing an inappropriate treatment with severe side effects, even deteriorating HRQOL during the last phase of life.

NSCLC patients with a targetable driver mutation (M+) are entitled to receive targeted therapy, which has been associated with higher response rates, fewer side effects, and longer progression-free survival compared to NSCLC patients without a targetable driver mutation (M−) [5]. Targeted therapy is usually less toxic than chemotherapy and immunotherapy. However, low-grade side effects are common and often chronic, which could also negatively impact a patient’s HRQOL [5,6,7,8]. HRQOL in NSCLC patients with and without a targetable driver mutation is thus likely to be different. Although assessing HRQOL is becoming increasingly important, data on differences in HRQOL in NSCLC patients with and without a targetable driver mutation are still limited [9]. 

To optimize clinical decision making for individualized treatment in patients with metastatic NSCLC, it is necessary to understand differences in HRQOL and survival outcomes between patients with and without a targetable driver mutation. Therefore, the aim of this study is to compare long-term HRQOL and survival in metastatic NSCLC patients with and without a targetable driver mutation. 

## 2. Materials and Methods

### 2.1. Study Design

The present study was conducted with prospectively collected data from the SYMPRO-lung study. SYMPRO-lung is a Dutch prospective, multicenter study in which the effect of online patient-reported outcome symptom monitoring on HRQOL in daily clinical practice is examined [10]. Monitoring is performed during and after lung cancer treatment. In short, patients are eligible if they have cytologically/histologically proven or radiologically suspect small or non-small cell lung cancer, are starting treatment (or a combination) with radiotherapy, surgery, chemotherapy, immunotherapy, and/or targeted therapy, are 18 years and older, have an Eastern Cooperative Oncology Group (ECOG) performance status [11] classification of 0, 1 or 2, and have access to the internet. Patients are excluded if they are already participating in a treatment study that includes structured symptom reporting, have a life expectancy of less than 15 weeks at time of inclusion, or either treatment or follow-up of the patient takes place in a center that does not participate in the study. Patients are still recruited from 3 academic and 11 non-academic hospitals in the Netherlands and provided informed consent at the start of treatment (up to 2 weeks after start of treatment). For this study, we included NSCLC patients in the control group with metastatic lung cancer, who were included between October 2019 and November 2020. The SYMPRO-lung study was approved by the Medical Ethical Committee (METc) of the VU University Medical Center in Amsterdam (12 July 2019 Trial NL7897) and is registered on the Dutch trial register trialregister.nl (Trial NL7897).

### 2.2. Demographic Characteristics

Demographic characteristics (education, employment status, marital status, and comorbidity) are reported by patients at baseline. Clinical data are reported by health care practitioners (HCPs) at baseline and include treatment characteristics (ECOG performance status, histological tumor type, and cancer stage according to the TNM 8th edition and (previous) treatment). During the follow-up assessments at 15 weeks, 6 and 12 months after start of treatment, the HCPs are asked to report ECOG performance status, current treatment, and treatment response (i.e., cured, complete or partial response, stable disease, progression, relapse, death, and/or other). 

### 2.3. Health-Related Quality of Life

HRQOL questionnaires are completed at baseline, 15 weeks (T1), and 6 months (T2) after the start of treatment. HRQOL was assessed with the European Organisation for Research and Treatment of Cancer Quality of Life Core Questionnaire (EORTC QLQ-C30) [12,13], which includes the overall (global health status and summary score) and functioning domains (physical functioning, role functioning, cognitive functioning, emotional functioning, and social functioning), and the symptom domains (fatigue, nausea and vomiting, pain, dyspnea, insomnia, appetite loss, constipation, diarrhea, and financial impact). For each domain, a summary score was calculated according to the EORTC manual [14]. Additionally, the QLQ-C30 summary score was also calculated as overall HRQOL by combining the 13 QLQ-C30 scale scores as the mean (excluding financial impact and a two-item global quality of life scale) [15]. All scores were linearly transformed to 0–100 scales. For the overall and functioning domains, a higher score reflects improved functioning, while for the symptom domains, a higher score reflects more symptomatology. Giesinger thresholds for clinical decline were also included to increase the interpretability of the QLQ-C30 questionnaire and make it more useful in daily clinical care [16]. A change of more than 10 points over time was considered a clinically relevant change in HRQOL [17]. 

### 2.4. Data Analysis

Patient characteristics are presented as proportions (for categorical variables) and mean (±SD) for continuous variables. Differences in baseline characteristics and HRQOL between patients with and without a targetable driver mutation were compared using independent samples *t*-tests (continuous variables) and chi-square tests (categorical variables). 

Generalized estimating equations (GEEs) were used to assess mean change scores in HRQOL over time between NSCLC patients with and without a targetable driver mutation. We constructed a model with a random intercept and an unstructured covariance structure. Improvement of fit of the models was compared based on the maximum likelihood fits. Differences in mean change scores over time between groups were interpreted with Cohen’s effect size (ES) [18]. An ES of 0.20 was considered small, 0.50 moderate and clinically significant, and 0.80 large [19].

Overall survival was defined as time from start of treatment to time of death from any cause, or to time of last follow-up (censoring). Progression-free survival (PFS) was defined as time from start of treatment until disease progression, tumor recurrence, or death from any cause. Cumulative incidence, 6-month, and 1-year mortality rates were investigated. Kaplan–Meier survival curves stratified for mutation status and clinically significant declines in HRQOL were plotted for both PFS and OS. All analyses were conducted using SPSS version 27. For all tests, two-sided *p* values ≤ 0.05 were considered statistically significant.

## 3. Results

### 3.1. Study Population

In total, 81 patients were included in this study, of whom 16 (20%) had a targetable driver mutation. Median follow-up duration was 15 months (IQR 11 to 17 months) with no difference between patients with and without a targetable driver mutation (16 vs. 15 months, respectively).

### 3.2. Baseline Characteristics

Table 1 shows baseline characteristics for the total study population and stratified for NSCLC patients with and without a targetable driver mutation. Mean age was 65.1 years and half of the patients (50.6%) were men. Most patients presented with ECOG performance status of 0 (33.3%) or 1 (59.3%) at baseline. Almost half of patients (44%) with a targetable driver mutation were treated with targeted therapy, whereas patients without a targetable driver mutation were most often treated with a combination of radiotherapy plus immunotherapy (43%). Except for molecular analysis and treatment, no further significant differences in baseline characteristics between the two patient groups were observed. M+ patients who received targeted therapy had a slightly better performance score compared to M− patients and M+ patients who did not receive targeted therapy. Furthermore, there were no significant differences between patients receiving targeted therapy and those who did not (Table S1).

### 3.3. Health-Related Quality of Life

Table 2 shows mean HRQOL values at baseline, 15 weeks, and 6 months of follow-up. At baseline, no significant differences in HRQOL between patients with and without a targetable driver mutation were observed. During follow-up, patients with a targetable driver mutation had significantly better overall HRQOL and social functioning compared to patients without a targetable driver mutation. Furthermore, patients with a targetable driver mutation had significantly less complaints of dyspnea, fatigue, nausea and vomiting, and constipation during follow-up (Table 2).

After adjustment for treatment, clinically relevant between-group differences in mean change scores over time were found for global HRQOL, physical functioning, and appetite loss (Table 3). At 15 weeks of follow-up, patients with a driver mutation had significantly better global HRQOL (mean difference 12.8, ES 0.61), physical functioning (mean difference 13.4, ES 0.63), and less appetite loss (mean difference 23.1, ES 0.73) compared to patients without a targetable driver mutation. Although HRQOL almost restored at 6 months of follow-up across all domains, patients with a driver mutation still showed significantly better physical functioning (mean difference 9.5, ES 0.45) and less appetite loss (mean difference 14.3, ES 0.45) compared to patients without a targetable driver mutation. No significant differences between the groups over time for the other HRQOL domains were observed.

### 3.4. Survival

**Figure 1:**
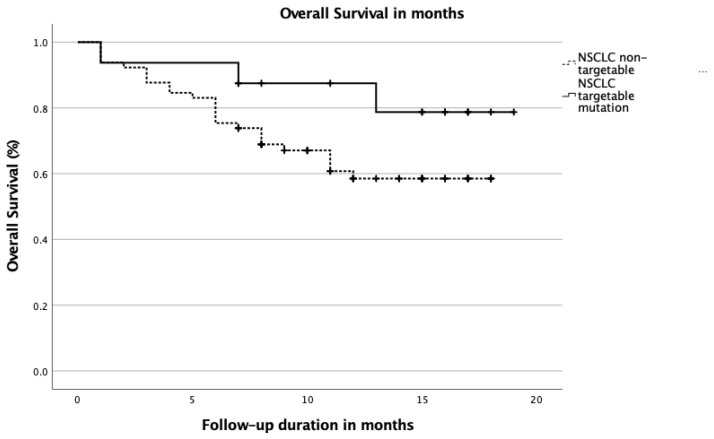
Kaplan–Meier survival curve stratified for NSCLC patients with and without a targetable driver mutation. Abbreviations; NSCLC; non-small cell lung cancer.

Table 4 shows OS rates and Figure 1 shows the Kaplan–Meier OS curve for the two groups. During follow-up, 28 (35%) patients died, which was not significantly different across the two patient groups. Mean OS was 14 months, not significantly different between the two groups (Table 4). Although non-significant, a trend towards better 12-month survival rates for patients with a targetable driver mutation compared to patients without a targetable driver mutation was observed (7% vs. 39%, *p*-value 0.074, respectively). No differences in PFS were observed.

### 3.5. HRQOL Decline

Since no significant differences in OS and PFS between the groups were observed, prognostic analyses for HRQOL declines on OS and PFS were performed for the entire patient group. At 6 months of follow-up, a clinically relevant decline (>10 points) in HRQOL (defined by the summary score) was significantly associated with both OS as well as PFS. Patients with a significant decline in HRQOL at 6 months of follow-up had a significantly shorter PFS (5 months vs. 12 months, *p*-value < 0.001) as well as a significantly shorter OS (11 months vs. 16 months, *p*-value 0.002).

## 4. Discussion

In this observational study of stage IV NSCLC patients, we found that patients with a targetable driver mutation had better HRQOL over time compared to NSCLC patients without a targetable driver mutation. Since we also observed a tendency towards favorable survival rates in patients with a targetable driver mutation, these patients are more likely to live longer with a better quality of life compared to those without a targetable driver mutation.

To our knowledge, this is the first observational study comparing HRQOL over time in stage IV NSCLC patients with and without a targetable driver mutation. An improved HRQOL in patients with a targetable driver mutation may not be surprising, given the improved HRQOL rates in targeted therapy trials [20,21]. Both survival rates and HRQOL may improve for the whole population of patients with lung cancer because of advancements in treatment options. An example is the addition of Metformin to treatment, a standard antidiabetic agent that shows promising results [22]. Nonetheless, not all patients with a targetable driver mutation received targeted therapy in this study (Table 1). Furthermore, even after correction for treatment, we observed better HRQOL over time in NSCLC patients with a targetable driver mutation compared to patients without a targetable driver mutation. Apart from less severe side effects, it could be that patients with a targetable driver mutation have less anxiety regarding their prognosis because their doctors may frame their mutation as relatively good news, resulting in favorable HRQOL over time [23]. Nonetheless, more research is needed to elucidate the mechanism behind the observed HRQOL benefit.

We did not observe differences in patient characteristics between patients with and without a targetable driver mutation. From the literature, we know that patients with a targetable driver mutation are more often younger, female, never-smokers, and of Asian ethnicity [24]. Although non-significant, patients with a targetable driver mutation in our study population were indeed slightly younger and more often female. Unfortunately, we did not have information on ethnicity or smoking history and can therefore not confirm the reported association between ethnicity, smoking, and mutational status.

In the total study population, patients with a clinically relevant decline in HRQOL over time had worse PFS and OS. This finding indicates that HRQOL in general is very important when it comes to individualized clinical decision making in stage IV NSCLC patients, regardless of their mutational status. For these patients, treatment is aimed at life extension while improving HRQOL. We recently found that pre-treatment HRQOL goals are only met by less than half of patients [3,4]. In addition, patients with an incurable disease often have a poor prognostic perception, as a result of the efficacy–effectiveness gap [25], (which can increase) deficient patient–doctor communication, as well as attempts of patients and their relatives to maintain the hope of a cure. Our results underline even more that taking HRQOL into account is essential to optimize clinical decision making for individualized treatment and thereby improve or at least maintain HRQOL in patients with stage IV NSCLC.

Strengths of this study include its novel focus on HRQOL over time in patients with and without a targetable driver mutation and the association of HRQOL declines with survival outcomes. There are also some limitations to our study. Because of the observational nature of the study design, no causal relationships can be distinguished. We had no information on cause-specific mortality and mutation type. Therefore, we cannot elucidate whether mortality was NSCLC-specific. Lastly, although our results are promising, the sample size was quite small and strict selection criteria were handled due to the study design. Therefore, our results might not be generalizable to ‘real world’ stage IV NSCLC patients who are generally older, and have a worse performance score and more comorbidities, including COPD, which may limit applicability of therapies.

## 5. Conclusions

In conclusion, our findings indicate that stage IV NSCLC patients with a targetable driver mutation have favorable HRQOL over time compared to stage IV NSCLC patients without a targetable driver mutation. Furthermore, clinically relevant HRQOL declines over time were significantly associated with worse survival, regardless of mutational status. These results indicate that HRQOL can play a potentially important role in clinical decision making and in shaping the patient’s expectations of prognosis. In addition, it can be of importance in individual risk stratification and tailored supportive follow-up care.

## Figures and Tables

**Table 1:** Baseline characteristics.

Characteristics	Total Study Population	NSCLC M+	NSCLC M−	*p*-Value
Total	81	16	65	
Age (years)	65.11 (9.46)	68.13 (10.21)	64.37 (9.20)	0.156
Gender (% male)	41 (50.6%)	6 (37.5%)	35 (53.8%)	0.276
*ECOG Performance Status*				0.142
0	27 (33.3%)	4 (25%)	23 (35.4%)	
1	48 (59.3%)	9 (56.3%)	39 (60%)	
2	6 (7.4%)	3 (18.8%)	3 (4.6%)	
*Comorbidities*				
Yes (%)	31 (38.3%)	7 (43.8%)	24 (36.9%)	0.775
*Histology*				<0.001
Adenocarcinoma, mutation unknown	16 (19.8%)	-	16 (24.6%)	
Adenocarcinoma, M−	33 (40.7%)	-	33 (50.8%)	
Adenocarcinoma, M+	16 (19.8%)	16 (100%)		
Squamous cell carcinoma	10 (12.3%)	-	10 (15.4%)	
Large cell carcinoma	6 (7.4%)	-	6 (9.2%)	
*Treatment plan*				<0.001
Immunotherapy	24 (29.6%)	3 (18.8%)	21 (32.3%)	
Immunotherapy combined	32 (39.5%)	4 (25%)	28 (43.1%)	
Radiotherapy	5 (6.2%)	1 (6.3%)	4 (6.2%)	
Chemotherapy combined	13 (16%)	1 (6.3%)	12 (18.5%)	
Targeted therapy	7 (8.6%)	7 (43.8%)	-	

Age is presented as mean (SD) and as *n* (%), other data are presented as *n* (%). Abbreviations: NSCLC, non-small cell lung cancer; M+, targetable driver mutation; ECOG, Eastern Cooperative Oncology Group.

**Table 2:** Mean Health-Related Quality of Life scores.

EORTC-QLQ-C30	All			NSCLC M−			NSCLC M+		
	T0 (*n* = 81)	T1 (*n* = 65)	T2 (*n* = 54)	T0 (*n* = 65)	T1 (*n* = 51)	T2 (*n* = 41)	T0 (*n* = 16)	T1 (*n* = 14)	T2 (*n* = 13)
*Functioning Domains*									
QLQ summary score	73.74 (15.09)	75.78 (13.80)	77.13 (13.86)	73.52 (15.65)	73.99 * (14.63)	75.93 (14.83)	74.63 (12.94)	82.30 * (7.48)	80.93 (9.78)
Global health status	60.29 (21.66)	63.21 (21.49)	66.36 (21.84)	60.51 (22.64)	60.62 * (22.67)	65.04 (23.44)	59.38 (17.71)	72.62 * (13.25)	70.51 (15.82)
Physical functioning	69.96 (19.89)	67.90 (21.85)	69.88 (21.15)	70.36 (19.76)	65.49 (22.73)	68.13 (21.47)	68.33 (21.01)	76.67 (16.07)	75.38 (19.89)
Role functioning	57.20 (31.06)	53.85 (27.29)	60.49 (26.56)	57.18 (30.76)	51.31 (28.45)	57.32 (27.90)	57.29 (33.32)	63.10 (20.86)	70.51 (19.43)
Emotional functioning	72.22 (18.63)	74.87 (19.68)	79.48 (16.57)	71.92 (18.08)	74.84 (20.51)	78.05 (17.75)	73.44 (21.35)	75.00 (16.98)	83.97 (11.52)
Cognitive functioning	81.28 (20.48)	83.59 (19.43)	79.94 (20.83)	80.51 (20.74)	83.00 (20.41)	77.64 (21.61)	84.38 (19.69)	85.71 (15.82)	87.18 (16.88)
Social functioning	70.99 (26.45)	68.21 (25.81)	73.46 (24.98)	68.97 (27.93)	63.73 * (26.61)	70.33 * (27.01)	79.17 (17.74)	84.52 * (13.81)	83.33 * (13.61)
*Symptom Domains*									
Fatigue	44.86 (25.91)	40.68 (26.23)	38.89 (25.33)	45.98 (27.21)	43.79 * (27.70)	40.38 (27.64)	40.28 (19.82)	29.37 * (16.08)	34.19 (16.01)
Nausea and vomiting	11.11 (20.92)	8.97 (15.05)	8.95 (18.23)	11.03 (20.04)	10.13 (16.36)	10.98 * (20.28)	11.46 (24.88)	4.76 (7.81)	2.56 * (6.26)
Pain	27.16 (28.31)	24.36 (27.65)	21.91 (24.41)	26.41 (28.09)	24.51 (27.76)	21.95 (25.94)	30.21 (29.95)	23.81 (28.28)	21.79 (19.70)
Dyspnea	32.10 (30.02)	32.82 (29.75)	32.72 (30.02)	33.33 (30.05)	36.60 * (30.00)	32.52 (28.37)	27.08 (30.35)	19.05 * (25.20)	33.33 (36.00)
Insomnia	30.04 (32.75)	17.95 (24.35)	18.52 (23.94)	28.18 (32.74)	19.61 (25.10)	18.70 (23.63)	35.42 (33.26)	11.90 (21.11)	17.95 (25.88)
Appetite loss	25.10 (32.29)	17.95 (24.35)	20.99 (31.25)	22.56 (31.24)	19.61 (25.97)	21.14 (33.96)	35.42 (35.42)	9.52 (15.63)	20.51 (21.68)
Constipation	16.46 (25.34)	13.33 (24.86)	9.88 (16.67)	18.46 (26.37)	15.69 * (26.96)	10.57 (15.70)	8.33 (19.25)	4.76 * (12.10)	7.69 (19.97)
Diarrhoea	6.17 (15.01)	7.69 (15.33)	8.64 (20.67)	6.67 (15.81)	6.54 (13.37)	8.13 (20.79)	4.17 (11.39)	11.90 (21.11)	10.26 (21.01)
Financial Difficulties	5.35 (15.33)	6.67 (14.67)	8.64 (17.35)	5.64 (15.10)	6.54 (14.94)	8.13 (17.92)	4.17 (16.67)	7.14 (14.19)	10.26 (16.01)

Scores indicate means (SD). EORTC Quality of life score and functioning domains: higher score = better QOL/functioning. EORTC Symptom Domains: higher score = worse symptoms. Abbreviations: T0, baseline; T1, 15 weeks; T2, 6 months; NSCLC, non-small cell lung cancer; M+, targetable driver mutation; * indicates significant differences (*p*-value < 0.05) between the M− and M+ group.

**Table 3:** Between-group differences in Health-Related Quality of Life between baseline to follow-up.

Quality of Life Domain	T0–T1 Between-Group Difference		T0–T2 Between-Group Difference	
	Mean Change (SE)	ES	Mean Change (SE)	ES
Global Quality of Life				
NSCLC M− (ref; M+)	−12.8 (20.9)	0.61	−5.9 (20.9)	0.28
Physical Functioning				
NSCLC M− (ref; M+)	−13.4 (21.3)	0.63	−9.5 (21.4)	0.45
Appetite loss				
NSCLC M− (ref; M+)	23.1 (31.8)	0.73	14.3 (31.2)	0.45

Scores are presented as mean change scores from baseline between groups, with NSCLC M+ as reference group. Abbreviations: T0, baseline; T1, 15 weeks; T2, 6 months; SE, standard error; ES, effect size; NSCLC, non-small cell lung cancer; M+, targetable driver mutation.

**Table 4:** Mortality rates.

Time Point	Total Study Population	NSCLC M−	NSCLC M+	*p*-Value
Total	81 (100%)	65 (80%)	16 (20%)	-
Total mortality	28 (35%)	25 (39%)	3 (11%)	0.240
6-month mortality (%)	17 (21%)	16 (25%)	1 (6%)	0.171
12-month mortality (%)	27 (33%)	25 (39%)	2 (7%)	0.074
Mean OS time (SE)	14.2 (0.75)	13.1 (0.79)	16.6 (1.3)	0.140

Data are presented as *n* (%). Abbreviations: NSCLC, non-small cell lung cancer; M+, targetable driver mutation; OS, overall survival; SE, standard error.

## Data Availability

Data available on request due to restrictions eg privacy or ethical. The data presented in this study are available on request from the corresponding author. The data are not publicly available due to privacy reasons from the participating patients.

## Supplementary Materials

The following are available online at https://www.mdpi.com/article/10.3390/cancers13174282/s1, Table S1: Baseline characteristics between patients with and without targeted therapy.
