# Supplementary material for: Health-Related Quality of Life and Survival in Metastasized Non-Small Cell Lung Cancer Patients with and without a Targetable Driver Mutation
Source: Cancers (Basel). 2021 Aug 25;13(17):4282. doi: 10.3390/cancers13174282 (PMC8428358; doi:10.3390/cancers13174282)
Supplement: Supplementary file 1 [file cancers-13-04282-s001.zip › cancers-1355035-SI.pdf]

Supplementary Materials: Health-Related Quality of Life and Survival in Metastasized Non-Small Cell Lung Cancer Patients with and without a Targetable Driver Mutation

Nicole E. Billingy, Vashti N. M. F. Tromp, Corina J. G. van den Hurk, Annemarie Becker-Commissaris and Iris Walraven

Table S1. Baseline characteristics between patients with and without targeted therapy.

**Characteristics**	**Total Study Population**	**NSCLC M+**	**NSCLC M+ Targeted**	**NSCLC M−**	***p*-value**
Total	81	9	7	65	
Age (years)	65.11 (9.46)	68.67 (10.12)	67.43 (11.1)	64.37 (9.20)	0.356
Gender (% male)	41 (50.6%)	4 (44.4%)	2 (28.6%)	35 (53.8%)	0.413
ECOG Performance Status					0.007
0	27 (33.3%)	-	4 (57.1%)	23 (35.4%)	
1	48 (59.3%)	9 (66.7%)	3 (42.9%)	39 (60%)	
2	6 (7.4%)	3 (33.3%)	-	3 (4.6%)	
Comorbidities					
Yes (%)	31 (38.3%)	7 (43.8%)		24 (36.9%)	
Histology					<0.001
Adenocarcinoma, mutation unknown	16 (19.8%)	-		16 (24.6%)	
Adenocarcinoma, M-	33 (40.7%)	-		33 (50.8%)	
Adenocarcinoma, M+	16 (19.8%)	9 (100%)	7 (100%)		
Squamous cell carcinoma	10 (12.3%)	-		10 (15.4%)	
Large cell carcinoma	6 (7.4%)	-		6 (9.2%)	
Treatment plan					<0.001
Immunotherapy	24 (29.6%)	3 (33.3%)		21 (32.3%)	
Immunotherapy combined	32 (39.5%)	4 (44.4%)		28 (43.1%)	
Radiotherapy	5 (6.2%)	1 (11.1%)		4 (6.2%)	
Chemotherapy combined	13 (16%)	1 (11.1%)		12 (18.5%)	
Targeted therapy	7 (8.6%)		7 (100%)	-	

Age is presented as mean (SD) and as *n* (%), other data are presented as *n* (%). Abbreviations: NSCLC Non-small cell lung cancer; M+ targetable driver mutation; M− no targetable driver mutation; ECOG Eastern Cooperative Oncology Group.
